# A General Framework for Modeling Sub- and Ultraharmonics of Ultrasound Contrast Agent Signals with MISO Volterra Series

**DOI:** 10.1155/2013/934538

**Published:** 2013-03-10

**Authors:** Fatima Sbeity, Sébastien Ménigot, Jamal Charara, Jean-Marc Girault

**Affiliations:** ^1^Université François Rabelais de Tours, UMR-S930, 37032 Tours, France; ^2^INSERM U930, 37032 Tours, France; ^3^IUT Ville d'Avray, Université Paris Ouest Nanterre La Défense, 92410 ville d'Avray, France; ^4^Département de Physique et d'Électronique, Faculté des Sciences I, Université Libanaise, Hadath, Lebanon

## Abstract

Sub- and ultraharmonics generation by ultrasound contrast agents makes possible sub- and ultraharmonics imaging to enhance the contrast of ultrasound images and overcome the limitations of harmonic imaging. In order to separate different frequency components of ultrasound contrast agents signals, nonlinear models like single-input single-output (SISO) Volterra model are used. One important limitation of this model is its incapacity to model sub- and ultraharmonic components. Many attempts are made to model sub- and ultraharmonics using Volterra model. It led to the design of mutiple-input singe-output (MISO) Volterra model instead of SISO Volterra model. The key idea of MISO modeling was to decompose the input signal of the nonlinear system into periodic subsignals at the subharmonic frequency. In this paper, sub- and ultraharmonics modeling with MISO Volterra model is presented in a general framework that details and explains the required conditions to optimally model sub- and ultraharmonics. A new decomposition of the input signal in periodic orthogonal basis functions is presented. Results of application of different MISO Volterra methods to model simulated ultrasound contrast agents signals show its efficiency in sub- and ultraharmonics imaging.

## 1. Introduction

 Medical diagnostic using ultrasound imaging was greatly improved with the introduction of ultrasound contrast agents. In ultrasound imaging, contrast agents are microbubbles [1]. Historically, the important difference between the acoustic impedance of the tissue and the gas encapsulated within the microbubbles was the first step to improve the contrast of echographic images. However, the contrast was still improved by taking into account the nonlinear behavior of microbubbles. In fact, when microbubbles were insonified by a sinusoidal excitation, they respond by generating harmonic components [2]. For example, second harmonic imaging (SHI) [3] consists in transmitting a signal at frequency *f*_0_ and receiving echoes at twice the transmitted frequency 2*f*_0_. However, harmonic generation during the propagation of ultrasound in the nonperfused tissue limits the contrast [4].

Many years ago, experimental studies have shown the existence of subharmonics at *f*_0_/2 [5] and ultraharmonics at ((3/2)*f*_0_, (5/2)*f*_0_,…) [6] in the microbubble response under specific conditions of frequency and pressure. The absence of these components in the backscattered signal by the tissue has enabled the introduction of sub- and ultraharmonics as an alternative of the harmonic imaging in order to enhance the contrast. Sub- and ultraharmonic imaging consists of transmitting a signal of frequency *f*_0_ and extracting components at *f*_0_/2, (3/2)*f*_0_, (5/2)*f*_0_,….

Many models have been developed to understand the dynamics of the microbubble [2]. Microbubble oscillation can be accurately described using models such as Rayleigh-Plesset modified equation [7–9]. However, to enable optimal separation of harmonic components, other nonlinear models like single-input single-output (SISO) Volterra model have been preferred [10]. A well known limitation of SISO Volterra model is its capacity to model exclusively harmonic components sub- and ultraharmonics are not modeled [11].

To overcome this difficulty, Boaghe and Billings [12] have proposed a multiple-input single-output (MISO) Volterra-based method. Input signals are specified by having sub-harmonic component at frequency *f*_0_/*N*. This approach has been applied in ultrasound medical imaging [13].

However, neither Boaghe and Billings [12] nor Samakee and Phukpattaranont [13] have clearly justified the required conditions to design a MISO Volterra decomposition able to model sub- and ultraharmonics.

To answer this untreated point, we propose a more general framework which firstly gives a clear justification regarding the choice of the model and secondly can offer interesting alternatives.

This paper is organized as follows: after recalling Volterra model and presenting the general framework of MISO Volterra methods, simulations of contrast ultrasound medical imaging followed by results are presented. Finally, a discussion completed by a conclusion closes the paper.

## 2. SISO Volterra Model

**Figure 1:**
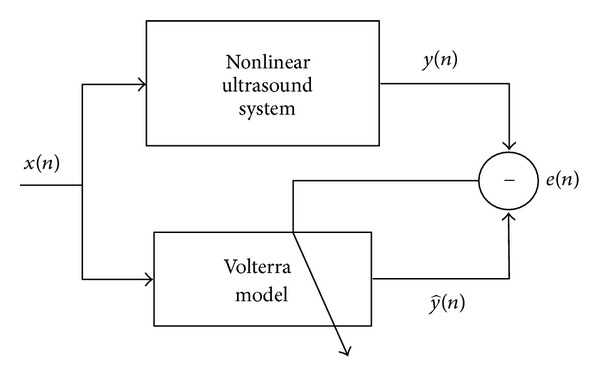
Block diagram of SISO Volterra model.

 Volterra series were introduced like Taylor series with memory [10]. Let *x*(*n*) and *y*(*n*) be, respectively, the input and the output signals in the discrete time domain *n* of the nonlinear system (see Figure 1). The output $\hat{y}(n)$ of Volterra model of order *P* and memory *M* is given in [14]. Note that, in our study focused on ultrasound imaging, a third-order Volterra model *P* = 3 is sufficient for the available transducers bandwidths. The output $\hat{y}(n)$ of SISO Volterra model of order *P* = 3 and memory *M* is given by

(1) y^(n)=h0+∑k1=0M−1h1(k1)x(n−k1)+∑k1=0M−1 ∑k2=0M−1h2(k1,k2)x(n−k1)x(n−k2)+∑k1=0M−1 ∑k2=0M−1 ∑k3=0M−1h3(k1,k2,k3)×x(n−k1)x(n−k2)x(n−k3),

where *h*_*p*_(*k*_1_, *k*_2_,…, *k*_*p*_) is the kernel of order *p* of the filter, with *p* ∈ {1,2, 3}.

Equation (1) could be rewritten as follows

(2) $$\begin{array}{c} y=X\cdot h, \end{array}$$

where the output signal is:

(3) $$\begin{array}{c} y={\left[y\left(M-1\right),y\left(M\right),\ldots ,y\left(L\right)\right]}^{T}, \end{array}$$

where *L* is the length of the signal *y*(*n*), and the vector of kernels is

(4) h=[h1(0),h1(1),…,h1(M−1),h2(0,0),  h2(0,1),…,h2(M−1,M−1),…,  hp(0,0,0),…,h3(M−1,M−1,M−1]T,

where the input matrix is

(5) $$\begin{array}{c} X={\left[x_{M-1},x_{M},\ldots ,x_{L}\right]}^{T}, \end{array}$$

with vector

(6) xn=[x(n),x(n−1),…,x(n−M+1),  x2(n),x(n)x(n−1),…,x2(n−M+1),  x3(n),x(n)x(n)x(n−1),…,  x3(n−M+1)]T,

with *n* ∈ {*M* − 1, *M*,…, *L*}.

The vector of kernels **h** is calculated to minimize the mean square error (MSE) between *y*(*n*) and $\hat{y}(n)$ according to the equation

(7) $$\begin{array}{c} \arg\;\underset{h}{\min}\left(𝔼\left[{\left(y\left(n\right)-\hat{y}\left(n\right)\right)}^{2}\right]\right), \end{array}$$

where *𝔼* is the symbol of the mathematical expectation.

Vector **h** is calculated using the least squares method

(8) $$\begin{array}{c} h={\left(X^{T}X\right)}^{-1}X^{T}y, \end{array}$$

if (**X** ^*T*^ **X**) is invertible. Otherwise, regularization techniques can be used.

Nevertheless, as reported in [12], it is not possible to model sub- and ultraharmonics with SISO Volterra model under this formulation. This is due to the fact mentioned in [12] that SISO Volterra model can only model frequencies at integer multiples of the input frequency.

To overcome this limitation, Boaghe and Billings [12] proposed a MISO Volterra-based solution and not any more a SISO Volterra. This point is discussed in Section 3.

## 3. General Framework of MISO Volterra Model

 According to Boaghe and Billings' claims [12], it is possible to model sub- and ultraharmonic components of the signal *y*(*n*) if the excitation signal to Volterra model has the sub-harmonic component at *f*_0_/*N*. The solution proposed by Boaghe and Billings [12] to show up the sub-harmonic component at frequency *f*_0_/*N* is to decompose the input signal *x*(*n*) into multiple input signals *x*_*i*_(*n*), each signal having frequency components at *f*_0_ and *f*_0_/*N*. From our point of view, Boaghe and Billings' approach [12] claimed two conditions that are intrinsically coupled by the choice of the decomposition method as follows:

(i) the input signal to Volterra model has sub-harmonic frequency component at *f*_0_/*N*;
(ii) Volterra system is a MISO system described by
  (9) $$\begin{array}{c} x\left(n\right)=\sum_{i=1}^{N}x_{i}\left(n\right). \end{array}$$

**Figure 2:**
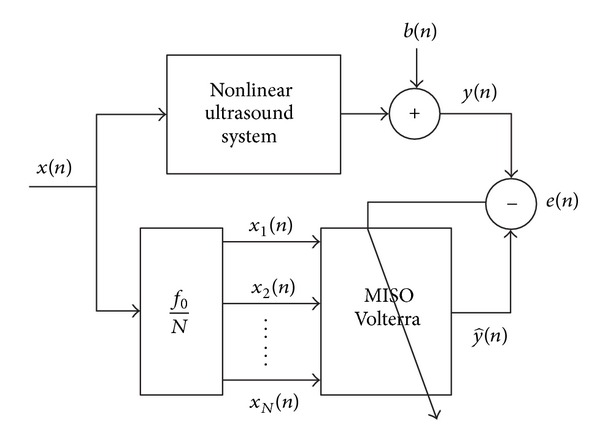
Block diagram of MISO Volterra model.

The block diagram of MISO Volterra model is presented in Figure 2.

A third condition that is not really explained in [12], however, it is a crucial condition to carry out this modeling procedure. It is the orthogonality condition between each multiple input of MISO Volterra model. Taking into account this third condition makes it possible to generalize Boaghe and Billings' approach presented in [12] as follows:

(10) $$\begin{array}{c} x\left(n,f_{0}\right)=\sum_{i=1}^{N}x_{i}\left(n,f_{0},N\right)=\sum_{i=1}^{N}\alpha_{i}\Psi_{i}\left(n,f_{0},N\right), \end{array}$$

where *α*_*i*_ are coefficients to be adjust and Ψ_*i*_(*n*, *f*_0_, *N*) is the periodic orthogonal basis functions having a spectral component at *f*_0_/*N*. Different bases could be proposed. In this study, two bases are presented as follows.

(1) In [12] a first periodic basis of orthogonal functions is proposed as follows:
  (11) Ψi(n,f0,N) =x(n,f0)∗∑k=−∞+∞Rect1/f0(nTs−kN+i−1f0),
  where *T*_*s*_ is the sampling period, ∗ represents the convolution product, and Rect_1/*f*_0__(*n*) is the rectangular function equal to 1 when −1/2*f*_0_ < *n* < −1/2*f*_0_ and equal to zero otherwise. Note that this approach is MISO1.
(2) In the present work, a new periodic basis of orthogonal functions is presented as follows:
  (12) Ψi(n,f0,N)=x(n,f0)+(−1)(i−1)×(x(n,f0)cos⁡(nTsw0N−1N)   +x~(n,f0)sin(nTsw0N−1N)),
  where $\tilde{x}(n)=ℋ\left(x(n)\right)$ is the Hilbert transform of *x*(*n*) and *w*_0_ = 2*πf*_0_. Note that this second is MISO2 approach.

For our application in contrast medical imaging, the sub-harmonic frequency is *f*_0_/2 [5–7], so *N* = 2.

As an illustration, when *x*(*n*) = *A*cos⁡ (*w*_0_ *nT*_*s*_) and *N* = 2, the decomposition is written:

(1) for the first basis, as follows:
  (13) x(n)=x1(n)+x2(n)=α1Ψ1(n,f0,2)+α2Ψ2(n,f0,2),
  with *α*_1_ = *α*_2_ = 1, and
  (14) Ψ1(n,f0,2)=Acos⁡(w0nTs)∗∑k=−∞+∞Rect1/f0(nTs−2kf0),Ψ2(n,f0,2)=Acos⁡(w0nTs)∗∑k=−∞+∞Rect1/f0(nTs−2k+1f0),
(2) and for the second basis, as follows:
  (15) x(n)=x1(n)+x2(n)=α1Ψ1(n,f0,2)+α2Ψ2(n,f0,2),
  with *α*_1_ = *α*_2_ = 1/2, and
  (16) Ψ1(n,f02)=Acos⁡(w0nTs)∗∑q=12δ(nTsq),Ψ2(n,f02)=Acos⁡(w0nTs)∗∑q=12(−1)(q−1)δ(nTsq),
  where *δ* (*n*) is the Dirac function. Finally, Ψ_1_ (*n*, *f*_0_/2) and Ψ_2_ (*n*, *f*_0_/2) can be simply rewritten as follows:
  (17) Ψ1(n,f0,2)=Acos⁡(w0nTs)+Acos⁡(w02nTs),Ψ2(n,f0,2)=Acos⁡(w0nTs)−Acos⁡(w02nTs).

**Figure 3:**
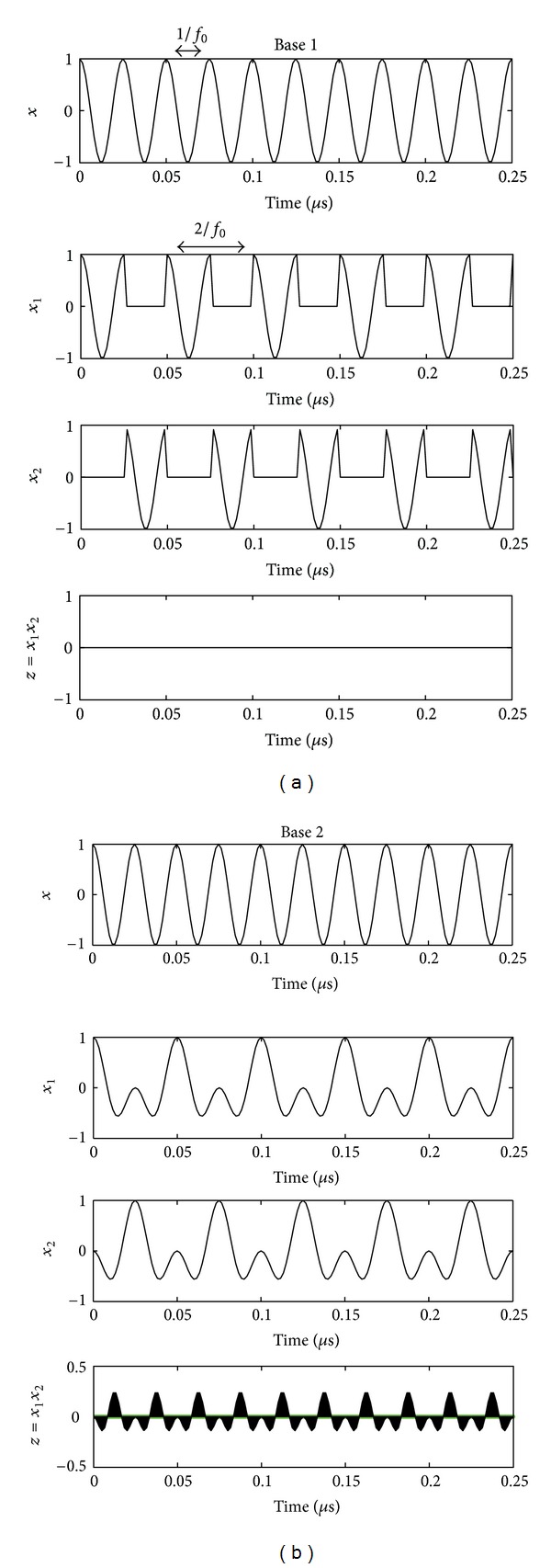
From top to bottom: input signal *x*, modified inputs *x*_1_, and *x*_2_, and the product *x*_1_ *x*_2_, (a) for the rectangular basis (basis 1) and (b) for the new basis (basis 2).

The two signals *x*_1_(*n*) and *x*_2_(*n*) for the two previous bases are represented in Figure 3.

It is obvious to show that for the two bases, the signals *x*_1_(*n*) and *x*_2_(*n*) are orthogonal because ∑*x*_1_(*n*)*x*_2_(*n*) = 0 (From a statistical point of view, the two signals *x*_1_(*n*) and *x*_2_(*n*) are orthogonal if and only if *𝔼*[*x*_1_(*n*)*x*_2_(*n*)] = 0. If *x*_1_(*n*) and *x*_2_(*n*) are stationary and ergodic, then *𝔼*[*x*_1_(*n*)*x*_2_(*n*)] = ∑(*x*_1_(*n*)*x*_2_(*n*)).). The algebraic area of the signal *z*(*n*) = *x*_1_(*n*)*x*_2_(*n*), shown in Figure 3, is equal to zero. 

Finally, if the components *x*_*i*_(*n*) are orthogonal to each other, then this also means that the output of Volterra model $\hat{y}(n)$ can be decomposed as follows:

(18) $$\begin{array}{c} \hat{y}\left(n\right)=\sum_{i=1}^{N}{\hat{y}}_{i}\left(n\right), \end{array}$$

where the components ${\hat{y}}_{i}(n)$ are also orthogonal to each other. A proof of this propriety is given in Appendix A.

**Figure 4:**
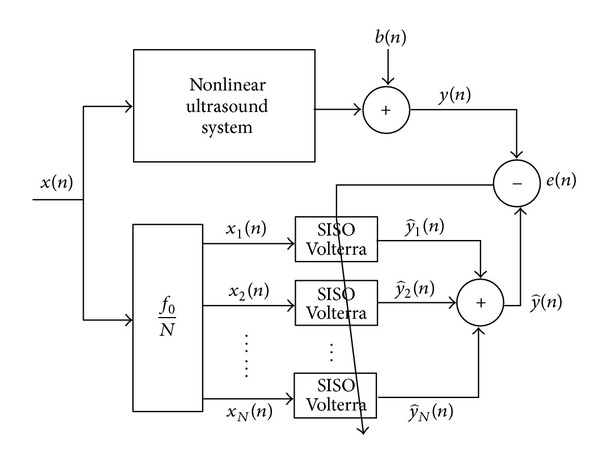
Block diagram of orthogonal MISO Volterra model.

The consequence of this statement is that MISO Volterra model can be considered as *N* parallel SISO Volterra models as depicted in Figure 4.

## 4. Simulations

 To validate the different proposed bases and to quantify its performances for application in contrast ultrasound medical imaging, realistic simulations are proposed. To carry out the simulations, the free simulation program B UBBLESIM developed by Hoff [7] was used to calculate the oscillations and scattered echoes for a specified contrast agent and excitation pulse. A modified version of Rayleigh-Plesset equation was chosen. The model presented by Church [15] and then modified by Hoff [7] is based on the theoretical description of microbubbles as air-filled particles with surface layers of elastic solids. In order to simulate the mean behavior of a microbubble cloud, we hypothesized that the response of a cloud of *N*_*b*_ microbubbles was *N*_*b*_ times the response of a single microbubble with the mean properties.

The incident burst to the microbubble is a sinusoidal wave of frequency *f*_0_ = 4 MHz (The resonance frequency of a microbubble of 1.5 *μ*m is about 2.25 MHz. Therefore, the emission frequency at 4 MHz is nearly the double of the resonance frequency.) To ensure the presence of sub- and ultraharmonics with moderate destruction of microbubbles, Forsberg et al. have proposed in [16] a pressure range from 1.2 MPa to 1.8 MPa. To limit the destruction of microbubbles, we set the pressure level to the lowest value at 1.2 MPa. The burst consists of 18 cycles. The sampling frequency is *f*_*s*_ = 60 MHz. The parameters of the microbubble are given in the Table 1 [13].

## 5. Results

 In this research, the performances of different modeling methods are evaluated qualitatively and quantitatively.

### 5.1. Qualitative Evaluation

**Figure 5:**
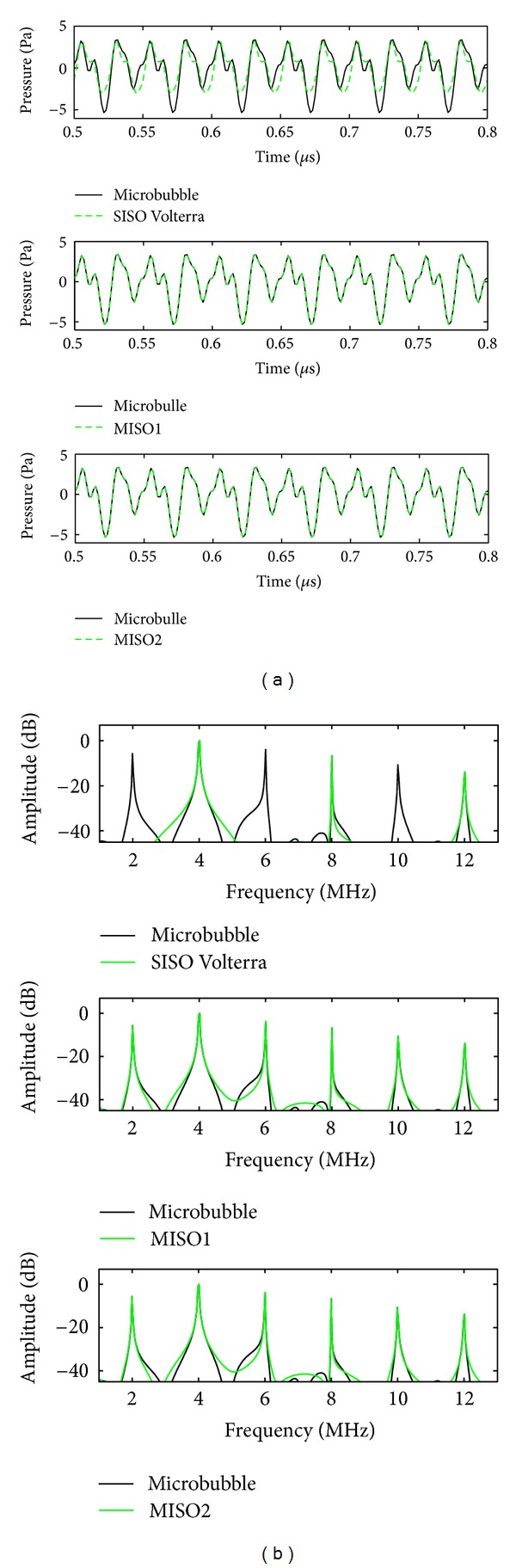
(a) Comparison between the backscattered signal by the microbubble *y*(*n*) (black) and its estimation $\hat{y}(n)$ (green): (top) the modeled signal with SISO Volterra model, (middle) MISO1 method, and (bottom) MISO2 method. (b) Spectra of different signals are presented in (1). Here SNR = *∞* dB, *P* = 3, and *M* = 19.

 To evaluate qualitatively the two MISO methods, MISO1 (with the basis proposed in [12]) and MISO2 (with the new basis proposed in the present work) with respect to SISO Volterra method, temporal representations of *y*(*n*) and $\hat{y}(n)$, and spectral representations |*Y*(*k*)|^2^ and $|\hat{Y}(k){|}^{2}$ of the nonlinear system backscattered by the contrast agent in nonlinear mode are presented in Figure 5.

Results presented in Figure 5 are obtained for a signal to tissue ratio SNR = *∞* and using Volterra model of order *P* = 3 and memory *M* = 19.

To better distinguish the different harmonic components of ultrasound signal, six cycles of 0.05 *μ*s are presented in Figure 5(a), and a bandwidth of 13 MHz covering the 3 harmonics potentially accessible in ultrasound imaging is presented in Figure 5(b). For both types of representations, the fundamental frequency, harmonics, sub- and ultraharmonics are well apparent. In Figure 5(a) (top), only harmonic components at *f*_0_, 2*f*_0_, and 3*f*_0_ are modeled by SISO Volterra. This result confirms that SISO Volterra system is unable to correctly model sub- and ultraharmonics at frequencies *f*_0_/2, (3/2)*f*_0_, and (5/2)*f*_0_. In Figure 5 (middle, bottom), all the spectral components are correctly modeled validating the two MISO approaches.

### 5.2. Quantitative Evaluation

**Figure 6:**
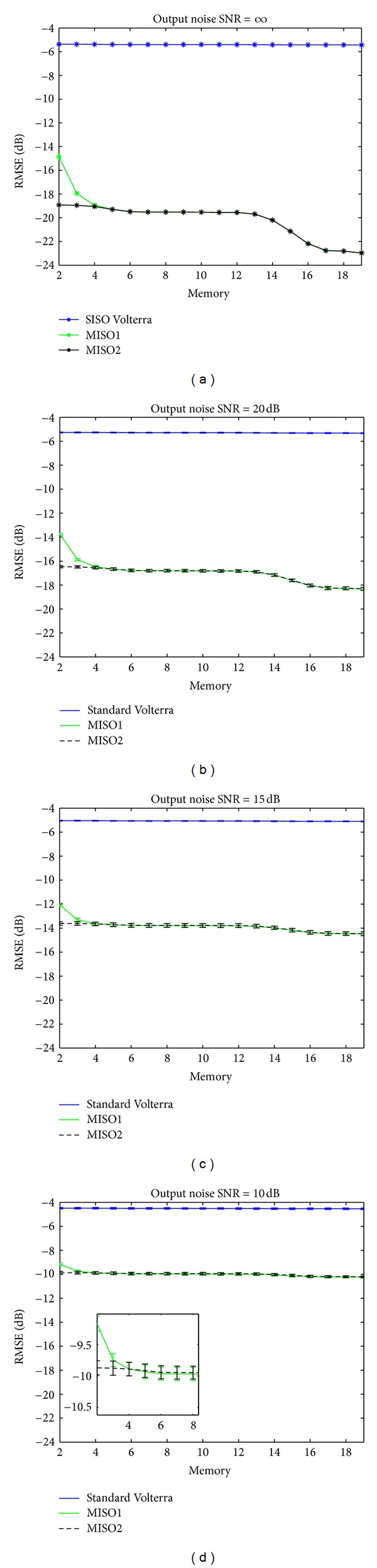
Variation of the RMSE in dB between the modeled signal with SISO Volterra (blue), MISO1 method (green), and MISO2 method (black) and the backscattered signal by the microbubble as a function of the memory of Volterra model in the presence of noisy output: (a) SNR = *∞* dB, (b) SNR = 20 dB, (c) SNR = 15 dB, and (d) SNR = 10 dB.

 To determine accurately the performances of the two methods and to know which Volterra approach provides the best performances a quantitative study is necessary. The relative mean square error (RMSE) defined as follows:

(19) $$\begin{array}{c} \text{RMSE}=\frac{𝔼\left[{\left(\hat{y}\left(t\right)-y\left(t\right)\right)}^{2}\right]}{𝔼\left[{\left(y\left(t\right)\right)}^{2}\right]} \end{array}$$

is evaluated for different noise levels at the system output. The noise level, adjusted as a function of SNR, is Gaussian and white. Ten realizations are made to evaluate the fluctuations of RMSE. RMSE for SNR = *∞*, 20,15, and 10 dB is reported in Figure 6. A zoom in Figure 6(d) shows the fluctuations of the EQMR around a mean value.

The main result of these simulations shows that regardless the SNR values, MISO Volterra methods provide a much better RMSE than SISO Volterra method. In fact, a gap between SISO Volterra method and the two methods MISO1 and MISO2 going from 5 to 16 dB can be obtained depending on the SNR conditions. These results confirm that SISO Volterra method is not suitable for sub- and ultraharmonic modeling. A zoom on Figure 6(d) emphasizes the small fluctuations of the RMSE. This result shows the robustness of the two MISO Volterra approaches towards noise.

Note that the RMSE obtained with the two MISO Volterra approaches are similar and follows the same trend. However, a small advantage in favor of MISO2 method with respect to MISO1 method for memory values *m* smaller than 6 is noted.

Finally, the more the memory increases, the more the RMSE decreases, indicating that the different methods tend asymptotically toward the optimal solution.

## 6. Discussions and Conclusions

 In the present research, we proposed a general framework that describes harmonic, sub-, and ultraharmonics modeling using Volterra decomposition. This framework allowed us to highlight three essential criteria instead of two, to accurately model sub- and ultraharmonics:

- as suggested in [12], the basis should be periodic of period *f*_0_/*N*;
- as suggested in [12], Volterra system should be a MISO system;
- as suggested in this work, the decomposition of the input signal to Volterra model *x*(*n*) must be done with an orthogonal basis.

This general framework has also justified the different steps of the decomposition thus allowing to propose new periodic orthogonal bases more efficient. It is the same for the choice of the order of Volterra model, which was limited to three. In fact, for more or less severe constraints on the ultrasound transducers bandwidth, the order can be reduced or increased.

This more general formulation provides a methodological basis for optimal sub- and ultraharmonics contrast imaging and opens a new research axis for more efficient periodic orthogonal bases of MISO Volterra systems and also for new MISO systems based on Hammerstein models or Wiener models.

## Figures and Tables

**Table 1:** The parameters of microbubbles [13].

Resting radius	*r*_0_ = 1.5 *μ*m
Shell thickness	*d*_Se_ = 1.5 nm
Shear modulus	*G*_*s*_ = 10 MPa
Shear viscosity	*η* = 1.49 Pa·s

## Appendix

### A. Decomposition of MISO Volterra Model of 2 Inputs to 2 SISO Volterra Models

A MISO Volterra model with *N* inputs is equivalent to *N* SISO Volterra models if and only if the mean square error to be minimized between *y*(*n*) and $\hat{y}(n)$ is the same in both cases. We will determine the conditions that must be satisfied by the inputs *x*_1_(*n*) and *x*_2_(*n*) of MISO Volterra when *N* = 2, to have this equivalence.

Volterra kernels are calculated using the least squares method by minimizing the mean square error (MSE) between *y*(*n*) and the modeled signal $\hat{y}(n)$:

(A.1) $$\begin{array}{c} 𝔼\left[{\left(y\left(n\right)-\hat{y}\left(n\right)\right)}^{2}\right]. \end{array}$$

For MISO Volterra model, the decomposition of *x*(*n*) into *x*_1_(*n*) and *x*_2_(*n*) such that *x*(*n*) = *x*_1_(*n*) + *x*_2_(*n*) requires that *y*(*n*) = *y*_1_(*n*) + *y*_2_(*n*). It follows that $\hat{y}(n)={\hat{y}}_{1}(n)+{\hat{y}}_{2}(n)$. The error to be minimized is

(A.2) 𝔼[(y(n)−y^(n))2] =𝔼[y(n)2]+𝔼[y^(n)2]−2𝔼[y(n)y^(n)] =𝔼[(y1(n)+y2(n))2]+𝔼[(y^1(n)+y^2(n))2]  −2𝔼[(y1(n)+y2(n))(y^1(n)+y^2(n))] =𝔼[y1(n)2]+𝔼[y2(n)2]+2𝔼[y1(n)y2(n)]  +𝔼[y^1(n)2]+𝔼[y^2(n)2]+2𝔼[y^1(n)y^2(n)]  −2𝔼[y1(n)y^1(n)]−2𝔼[y1(n)y^2(n)]  −2𝔼[y2(n)y^1(n)]−2𝔼[y2(n)y^2(n)].

For the 2 SISO Volterra models of inputs *x*_1_(*n*) and *x*_2_(*n*) and outputs *y*_1_(*n*) and *y*_2_(*n*), respectively, the error to be minimized is

(A.3) 𝔼[(y1(n)−y^1(n))2]+𝔼[(y2(n)−y^2(n))2] =𝔼[y1(n)2]+𝔼[y^1(n)2]−2𝔼[y1(n)y^1(n)]  +𝔼[y2(n)2]+𝔼[y^2(n)2]−2𝔼[y2(n)y^2(n)].

A MISO Volterra model could be seen as 2 SISO Volterra models if (A.2) and (A.3) are equal. This equality gives

(A.4) 𝔼[y1(n)y2(n)]+𝔼[y^1(n)y^2(n)] −𝔼[y1(n)y^2(n)]−𝔼[y2(n)y^1(n)]=0.

One possible solution is that each term of the equation is equal to zero:

(A.5) 𝔼[y1(n)y2(n)]=0,𝔼[y^1(n)y^2(n)]=0,𝔼[y1(n)y^2(n)]=0,𝔼[y2(n)y^1(n)]=0.

Therefore

(A.6) y1(n)⊥y2(n),y^1(n)⊥y^2(n),y1(n)⊥y^2(n),y2(n)⊥y^1(n),

where ⊥ means orthogonal. Elsewhere, ${\hat{y}}_{1}(n)$ and ${\hat{y}}_{2}(n)$ are calculated according to (1). That implies that

(A.7) 𝔼[y^1(n)y^2(n)] =𝔼[∑k1=0M−1h1(k1)x1(n−k1)∑k1=0M−1h1′(k1)x2(n−k1)   +∑k1=0M−1 ∑k2=0M−1h2(k1,k2)x1(n−k1)x1(n−k2)   ×∑k1=0M−1 ∑k2=0M−1h2′(k1,k2)x2(n−k1)   ×  x2(n−k2)+⋯]=0.

One possible solution is that each term of the equation is equal to zero. For the first term, we get

(A.8) 𝔼[∑k1=0M−1h1(k1)x1(n−k1)∑k1=0M−1h1′(k1)x2(n−k1)] =∑k1=0M−1h1(k1)h1′(k1)𝔼[x1(n−k1)x2(n−k1)] =0.

The last equation implies that *x*_1_(*n*)⊥*x*_2_(*n*). For the other terms in (A.7), we obtain the same conclusion *x*_1_(*n*)⊥*x*_2_(*n*). Therefore, ${\hat{y}}_{1}(n)$ and ${\hat{y}}_{2}(n)$ are orthogonal if *x*_1_(*n*) and *x*_2_(*n*) are also orthogonal.

Elsewhere, if ${\hat{y}}_{1}(n)$ and ${\hat{y}}_{2}(n)$ are the estimations of *y*_1_(*n*) and *y*_2_(*n*), then $\hat{y}(n)\approx y_{1}(n)$ and ${\hat{y}}_{2}(n)\approx y_{2}(n)$. This means that the orthogonality of ${\hat{y}}_{1}(n)$ and ${\hat{y}}_{2}(n)$ implies the orthogonality of each couple formed by the four signals presented in (A.5). This is true if and only if *x*_1_(*n*)⊥*x*_2_(*n*).

Therefore, a MISO Volterra model with two inputs could be treated as two SISO Volterra models if the two inputs are orthogonal. This result could be generalized for MISO Volterra model with *N* inputs.
